# Irritation of a Branch of the Trigeminus Nerve at Its Central Extremity

**Published:** 1841-04

**Authors:** 

**Affiliations:** Altenkirchen


*Irritation of a Branch of the Trigeminus Nerve at its central extremity.* By Dr. Budge, of Altenkirchen. — A peasant woman, forty-six years old, was affected for the first time in her sixth year with a severe periodical pain over the right eye, for which she could ascribe no other cause than exposure to cold and damp one winter's day. Previously she had always been healthy, and her parents attained a very advanced age. The pain continued from her sixth to her twelfth year, recurring about every fortnight, lasting from twenty-four to thirty hours each time, and then leaving her again well and hearty. When she was twelve years old it ceased; and during the next twelve years never once returned. In the interval she commenced and regularly continued menstruating. But at the end of this time when twenty-four years old, she had a child, and eight days after delivery her old pain returned; from that time, now twenty-two years ago, she has never been a week without it.

The pain usually begins at midnight, awakening the patient from sleep, and lasts to the same time in the second night following, i. e. for about forty-eight hours, It is preceded and accompanied by chilliness, which at last terminating in sweat, indicates the approach of the end of the attack. The pain extends from the lamdoidal suture of the right side, and is most severe at the posterior and anterior surfaces of the upper eyelid, about half an inch above the brow, and a short distance below the eye of the same side. It is a stabbing pain, and so intense that the mere thought of it fills the patient with horror; it continues uninterruptedly through the whole period, and shortly before it ceases attains an indescribable severity. The slightest touch excites intense agony in the affected part. The immediate neighborhood and all the rest of the face are as to sensation perfectly natural; but the features on the right side are less marked than on the left side of the face. The cornea (during the pain) is rather turbid, and the eye is filled with tears, the sclerotica is reddened, and the vessels of the conjuctiva turgid; the tarsi are also swollen and red. The whole eye is turned outwards, so that the cornea is nearly hidden; and the patient can with difficulty look straigt forward with it, partly from loss of power over it, and partly because the straight position, if retained but for a few instants, excites giddiness and vomiting.

Soon after the commencement of the pain, giddiness comes on, and compels the patient to keep her bed; it increases with the pain, and usually lasts a day longer than it does. The patient is at the same time constantly tormented by sickness and vomiting of food, mucus, and bile. Her tongue is quite clean and her bowels regular, but she has no appetite. The organs of the abdomen and chest and the pulse are in a normal condition.

The first and second cervical vertebræ are a little sensitive on pressure, and from the third to the second dorsal there is extreme sensibility, especially over the third, fourth, fifth, and sixth cervical. As often as these are struck the patient flinches and complains of pain in the right eye, and immediately after vomits. The right arm also which sometimes moves spontaneously when the pain is very intense, is sometimes convulsed when these vertebræ are pressed.

When the paroxysm is about to cease, the intensity of the pain, the squinting and the giddiness decrease, and soon after a general sweat comes on. At the last the patient sneezes once or twice (and always, she says, through the right nostril) and then, except for the giddiness, which lasts a day longer, the disease is for the time removed. Every fresh attack, however, is severer than the preceding, in respect both of the pain and of the squinting. The paroxysms are rarer in the summer than in the winter and spring; mental anxiety and passion accelerate and aggravate them, but neither menstruation nor pregnancy has any influence on them.

In the intervals between the attacks of pain, there is no external appearance of illness whatever, except a slight outward squint; and, but for the influence of pressure on the spine, the woman might be deemed perfectly healthy. By this, however, though there is never any spontaneous pain in the back, all the signs of the disease may be in a slight degree voluntarily reproduced; as soon as the lower cervical vertebræ are pressed, the patient complains of the pain of her eye and head, the right eye turns outwards, tears flow into it, and sickness or even vomiting comes on. And again, all these phenomena cease as soon as the pressure is remitted.

Of the above singular symptoms, the author offers the following explanations:

The first and most important, the pain over the right eye, is evidently the result of a state of irritation of the majority of the fibres of the frontal branch of the trigeminus, whose exact course it follows; for the patient says that the pain is not in the eye itself, but passes above it from behind forwards, and extends upwards and a little inwards towards the nose. The pain in the back further shows that the exact seat of this irritation is the central extremity of the nerve, of which, as is well known, the roots have been traced to the region of the third and fourth cervical vertebræ and probably go down still lower. Immediately over this part, the patient has pain on pressure: and by it the pain in the eye may be produced and increased at will, although, except during the paroxysms, no more pain is excited by pressure on the right than by that on the left eye.

The pain in the other adjacent vertebræ probably results both from the stimulus of pressure on the extremities of the nerves over them, which from their proximity to the nervous centre are especially liable to exalted excitability, and from the immediate effects of the pressure on the membranes which are themselves excited and sensitive in an unnatural degree.

The drawing of the eye outwards is the result of an influence on the sixth nerve; an influence reflected from the sensitive nerves. It is not probable that the central ends of the fibres of the sixth are directly irritated; for if that were the case, the same extremities of the other nervous fibres that lie between the sixth and the fifth would most likely be also irritated, and the morbid phenomena would be more extensive. The twitchings of the right arm may also in like manner be regarded as reflex motions.

The organic alterations in the eye must be considered as signs of the excitement of the trigeminus; and as when a sensitive nerve is divided, nutrition is materially impaired, and the part it supplies undergoes a kind of maceration, so when one is irritated the same part is inflamed.

The giddiness is thus explicable; fibres of the trigeminus may be traced into the crura cerebelli ad frontem, near the spot from which the nerve comes out, and as Magedie has shown that when these crura are divided, the balance of the body seems to be lost, it is clear how an irritation of the trigeminus induces giddiness, and how that symptom is most marked when the signs of irritation are passing off or gone.

The vomiting is explained partly by the affection of the spinal cord and partly by the influence of the nerves on the sight. (In explanation of this the author refers to his own investigations on vomitings recently published.)

The shivering is the result of the stimulus of the central extremities of the sensitive nerves in the spinal cord; and it is comparable with the results of several of the author's experiments, in which he has observed that on exposing the spinal cord in a dog or other animal, and gently stroking it with a feather, all the characters of a shivering fit are immediately produced. In this, as in all other cases, the condition of the central extremity of the nervous fibre is felt as if it were that of its peripheral extremity.

The sneezing at the end of the paroxysms probably results from the nasal branch of the trigeminus becoming involved in the excitement when the irritation of the frontal is at its greatest height. The excitement is in this case also probably communicated in the nervous centre, though felt where the fibres are distributed; and it is communicated not to the sensitive nerves alone, but by reflex action to the nerves of the respiratory muscles.

The author, believing that the esential cause of the symptoms was a congestion of the vessels of the frontal nerve, had ten cupping-glasses applied every eight days near the vertebræ at which pain was produced by pressure; these were applied three times; mercurial ointment was rubbed in morning and evening, and four grains of sulphate of quinine with two of extr. nuscis vomicæ were given in two doses every day. Their result was most strking; the patient, who had for twenty-two years been entirely unrelieved by a variety of methods of treatment, had no severe attack after this was employed; after several months had elapsed, she continued well, and even the divergence of the right eye was in some degree lessened.—*Brit. and For. Rev., from Casper's Wochenschrift. Oct.* 3, 1840.—*Med. Examiner, Feb.* 27, 1841.

